# Complete Genome Sequence of a Bovine Viral Diarrhea Virus Subgenotype 1e Strain, SLO/2407/2006, Isolated in Slovenia

**DOI:** 10.1128/genomeA.01310-16

**Published:** 2016-11-17

**Authors:** Ivan Toplak, Urška Kuhar, Darja Kušar, Bojan Papić, Simon Koren, Nataša Toplak

**Affiliations:** a Institute of Microbiology and Parasitology, Veterinary Faculty, University of Ljubljana, Ljubljana, Slovenia; b Omega d.o.o., Ljubljana, Slovenia

## Abstract

Bovine viral diarrhea virus (BVDV) subgenotype 1e was isolated for the first time in Slovenia in 2006. Here, we report the complete genome sequence of BVDV-1e, strain SLO/2407/2006. The published genome will increase our understanding of the molecular characteristics of the BVDV-1e strains circulating in Europe.

## GENOME ANNOUNCEMENT

Bovine viral diarrhea virus (BVDV) is an important pathogen of ruminants that causes acute and persistent infections throughout the world (1), which results in severe economic losses in the cattle industry (2). The two species BVDV-1 and BVDV-2 are part of the genus *Pestivirus* in the family *Flaviviridae*. BVDV-1 is heterogeneous and further divided into 11 to 16 subgenotypes (3, 4). In Slovenia, monitoring of the BVDV infections has been ongoing since 1994 (5). In our previous study, it was discovered that four subgenotypes of BVDV (1b, 1d, 1f, and 1g) were circulating in the infected cattle farms between 1997 and 2004 (6). In 2006, an acute infection with BVDV was confirmed in a group of 28 cattle imported from France. BVDV was confirmed by reverse transcription-PCR (RT-PCR) from serum samples from the diseased animals and was successfully isolated on a bovine turbinate cell line. Genotyping of the 5′ untranslated region (UTR) and N^pro^ identified for the first time the presence of BVDV subgenotype 1e strains in Slovenia. Within the voluntary control program, the subgenotype BVDV-1e has also been detected in 2014 in two additional herds (7).

Total RNA extraction was performed from the BVDV isolate SLO/2407/2006 using the QIAamp viral RNA kit (Qiagen, Hilden, Germany). For sequencing the complete BVDV genome, an RNA library was prepared using the Ion Total RNA sequencing kit version 2, and the enrichment was carried out using the Ion PGM Template OT2 200 kit (Ion Torrent; Thermo Fisher Scientific, Carlsbad, CA, USA). The amplified library was sequenced on the Ion PGM platform using the Ion PGM HiQ sequencing kit and Ion 314 Chip version 2 (Ion Torrent; Thermo Fisher Scientific). Sequenced Ion Torrent reads were quality checked and trimmed using Ion Torrent Suite version 5.0.2 and assembled into contigs by *de novo* assembly using the Genome Sequencer software version 2.9 (Roche).

The complete genome of the BVDV strain SLO/2407/2006 comprises 12,258 nucleotides (nt), with 5′ and 3′ UTRs of 377 nt and 185 nt, respectively. The single large open reading frame codes for 3,898 amino acids. Compared to the published complete sequences of BVDV in GenBank, SLO/2407/2006 is rather heterogeneous, displaying only 74% to 85% nucleotide homology. The closest sequence with 85% homology is strain Carlito from Switzerland, belonging to the subgenotype BVDV-1e (8). The previously published 5′ UTR sequences of subgenotype BVDV-1e determined in France, Switzerland, Austria, Czech Republic, Denmark, Italy, Portugal, Slovakia, Spain, Germany, and the United Kingdom (3, 8–19) have 96% and 97% nucleotide identity with the 5′ UTR of SLO/2407/2006, confirming the wide distribution of this strain in Europe. The genetically closely related strain LV/LF15/12 (accession no. KP715120) was identified in 2012 also in Brazil. Only very limited data are available for the full-length BVDV-1 isolates other than 1a and 1b. Therefore, the publication of the second full-genome BVDV-1e sequence represents an important contribution in filling this gap.

### Accession number(s).

The complete genome sequence of BVDV strain SLO/2407/2006 has been deposited to GenBank under accession number KX577637.
